# Plant Phenotypic and Transcriptional Changes Induced by Volatiles from the Fungal Root Pathogen *Rhizoctonia solani*

**DOI:** 10.3389/fpls.2017.01262

**Published:** 2017-07-21

**Authors:** Viviane Cordovez, Liesje Mommer, Kay Moisan, Dani Lucas-Barbosa, Ronald Pierik, Roland Mumm, Victor J. Carrion, Jos M. Raaijmakers

**Affiliations:** ^1^Department of Microbial Ecology, Netherlands Institute of Ecology (NIOO-KNAW) Wageningen, Netherlands; ^2^Laboratory of Phytopathology, Wageningen University Wageningen, Netherlands; ^3^Plant Ecology and Nature Conservation Group, Wageningen University Wageningen, Netherlands; ^4^Laboratory of Entomology, Wageningen University Wageningen, Netherlands; ^5^Plant Ecophysiology, Institute of Environmental Biology, Utrecht University Utrecht, Netherlands; ^6^Wageningen Plant Research, Business Unit Bioscience, Wageningen University and Research Wageningen, Netherlands; ^7^Centre for Biosystems Genomics Wageningen, Netherlands; ^8^Institute of Biology, Leiden University Leiden, Netherlands

**Keywords:** fungal volatiles, plant growth promotion, plant resistance, plant transcriptome, auxin

## Abstract

Beneficial soil microorganisms can affect plant growth and resistance by the production of volatile organic compounds (VOCs). Yet, little is known on how VOCs from soil-borne plant pathogens affect plant growth and resistance. Here we show that VOCs released from mycelium and sclerotia of the fungal root pathogen *Rhizoctonia solani* enhance growth and accelerate development of *Arabidopsis thaliana*. Seedlings briefly exposed to the fungal VOCs showed similar phenotypes, suggesting that enhanced biomass and accelerated development are primed already at early developmental stages. Fungal VOCs did not affect plant resistance to infection by the VOC-producing pathogen itself but reduced aboveground resistance to the herbivore *Mamestra brassicae*. Transcriptomics of *A. thaliana* revealed that genes involved in auxin signaling were up-regulated, whereas ethylene and jasmonic acid signaling pathways were down-regulated by fungal VOCs. Mutants disrupted in these pathways showed similar VOC-mediated growth responses as the wild-type *A. thaliana*, suggesting that other yet unknown pathways play a more prominent role. We postulate that *R. solani* uses VOCs to predispose plants for infection from a distance by altering root architecture and enhancing root biomass. Alternatively, plants may use enhanced root growth upon fungal VOC perception to sacrifice part of the root biomass and accelerate development and reproduction to survive infection.

## Introduction

Plants interact with a multitude of (micro)organisms, including beneficial symbionts, fungal pathogens and insects. In the chemical interplay between microorganisms and plants, a wide range of compounds play a significant role. In this context, specific emphasis is given to volatile organic compounds (VOCs). This chemically diverse group of compounds can travel longer distances than other metabolites, facilitating a multitude of interactions with other organisms both below- and aboveground (Schulz and Dickschat, 2007; Das et al., 2012; Junker and Tholl, 2013). For example, during insect herbivore attack, plants trigger the emission of VOCs to attract natural enemies of the herbivores and to elicit resistance mechanisms in neighboring plants (Paré and Tumlinson, 1999; Dicke and Loreto, 2010; Mumm and Dicke, 2010). In addition, plants use VOCs during resource limitation to detect the presence of proximate competitors, such as neighboring plants, and to inhibit the competitors’ growth and development (Kegge and Pierik, 2010).

Plants also perceive VOCs emitted by other (micro)organisms present in their environment. It is now well established that VOCs emitted by beneficial soil microorganisms mediate, from a distance, interactions with plants and other microorganisms (Bailly and Weisskopf, 2012; Effmert et al., 2012; Bitas et al., 2013; Schmidt et al., 2015). VOCs emitted by plant-associated bacteria have been reported to promote plant growth, to induce plant systemic resistance, and to affect motility and antibiotic resistance in other bacteria (Ryu et al., 2003, 2004; Lee et al., 2012; D’Alessandro et al., 2014; Park et al., 2015). Fungal VOCs are also widespread in nature, but less well studied than bacterial VOCs. To date, approximately 250 fungal VOCs have been described, including acids, alcohols, aldehydes, esters, short-chain fatty acids, lipid oxides, terpenes and phenolics (Morath et al., 2012; Roze et al., 2012). The most well-known fungal VOC is 1-octen-3-ol, also referred to as the “mushroom smell,” which functions as a developmental signal for several fungal species (Chitarra et al., 2004; Herrero-Garcia et al., 2011).

To date, information on the ecological functions of fungal VOCs is limited and fragmented, in particular for VOCs produced by soil-borne plant pathogenic fungi. Hung et al. (2013) showed that VOCs emitted by the mycoparasitic fungus *Trichoderma viride* increased biomass and chlorophyll content of *Arabidopsis thaliana* seedlings. In contrast, Kottb et al. (2015) showed that VOCs emitted by *T. asperellum* inhibited plant growth and increased the levels of the plant hormones salicylic and abscisic acid, resulting in enhanced resistance against pathogenic fungi. Bitas et al. (2015) further showed that VOCs emitted by *Fusarium oxysporum* promoted the growth of *A. thaliana* and *Nicotiana tabacum*, and affected auxin transport and signaling. A more recent study showed that VOCs emitted by *Alternaria alternata* enhanced growth, early flowering and photosynthesis rates of *A. thaliana*, maize and pepper by affecting the levels of plastidic cytokinin (Sanchez-Lopez et al., 2016). Collectively, these few studies suggest that VOCs from plant-associated fungi may modulate the trade-off between plant growth, development and resistance. However, the natural functions of fungal VOCs as well as the underlying molecular mechanisms induced in plants remain largely unknown.

Here, we investigated the short- and long-term effects of VOCs emitted by the soil-borne plant pathogenic fungus *Rhizoctonia solani* on growth, development and resistance of *A. thaliana*. We exposed seeds and seedlings to the fungal VOCs and investigated their impact on seed germination, shoot and root biomass, and resistance belowground to the VOC-producing fungus as well as aboveground to the generalist herbivore *Mamestra brassicae*. As a first step to understand the underlying molecular mechanisms, we performed genome-wide transcriptome analysis of *A. thaliana* seedlings exposed to *R. solani* VOCs and subsequently tested a range of *A. thaliana* mutants disrupted in genes involved in ethylene and auxin pathways. The putative natural functions of the VOCs in pathogen–plant interactions are discussed.

## Materials and Methods

### Plants, Fungi, and Insect

*Arabidopsis thaliana* wild-type Col-0 was obtained from the collection of the Department of Phytopathology at Wageningen University, the Netherlands. Other genotypes used were the ethylene-insensitive mutants *etr1-4* (Chang et al., 1993) and *ein 3eil1* (Alonso et al., 2003), the auxin receptor mutant *tir1 afb1*, the auxin biosynthesis mutant *wei8* [similar to *sav3*, (Stepanova et al., 2008)], the PHYTOCHROME INTERACTING FACTOR (PIF) double mutant *pif4 pif5* (Lorrain et al., 2008). For the negative PIF regulator (HFR1) both the knockout mutant *hfr1-5* (Sessa et al., 2005) and the stable overexpressor p35S:*G-BH-03* (Galstyan et al., 2011) were used. Seeds were surface sterilized as previously described (van de Mortel et al., 2012) and kept in the dark at 4°C for three to 4 days before sowing. Plates and pots containing plants were grown in climate cabinets (21°C; 180 μmol light m^-2^ s^-1^ at plant level; 16 h : 8 h, light : dark; 60–70% R.H.).

The fungus *R. solani* AG2-2 IIIB was obtained from the collection of the Sugar Beet Research Institute, Bergen op Zoom, the Netherlands. Fungal cultures were started with a mycelial plug (Ø 5 mm) on 1/5^th^ strength Potato Dextrose Agar (1/5^th^ PDA, Oxoid) at 25°C. Sclerotia were obtained from a fungal culture incubated on 1/5^th^ PDA at 25°C for 3 weeks.

The generalist insect herbivore *M. brassicae* L. (Lepidoptera: Noctuidae; cabbage moth) was reared on *Brassica oleracea* L. var. gemmifera cv. Cyrus in a controlled growth chamber (22 ± 2°C; 16 h: 8 h, light: dark; 40–50% R.H.). Newly-emerged larvae were used in the experiments.

### Effects of Fungal Volatiles on Plant Growth and Development

**FIGURE 1:**
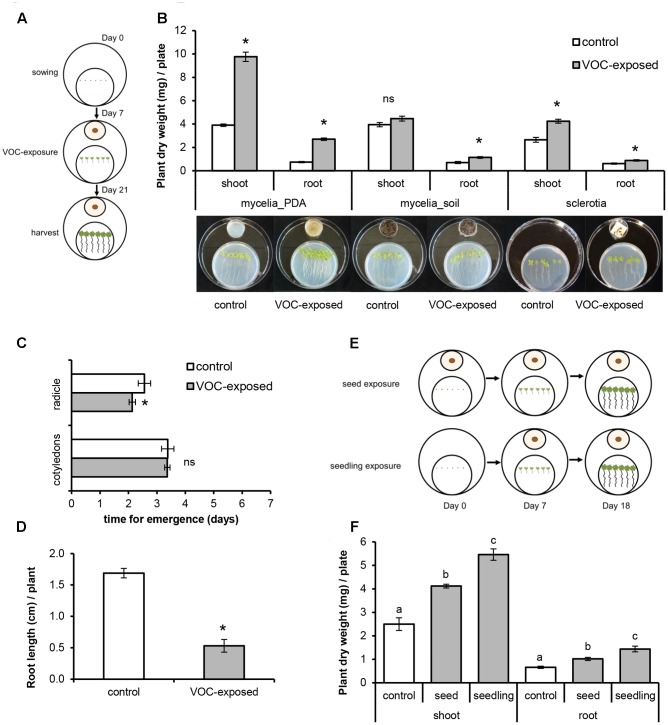
Effects of volatile organic compounds (VOCs) produced by *Rhizoctonia solani* on growth and development of *Arabidopsis thaliana*. Plants exposed to the fungal VOCs are referred as ‘VOC-exposed’ whereas plants exposed to agar medium or soil-sand mixture are referred as ‘control.’ Each biological replicate consists of one Petri dish with six seedlings each. Asterisks indicate statistically significant differences based on pairwise comparisons between VOC-exposed and control plants (Student’s *t*-test or Mann–Whitney *U* test, *P* < 0.05). Non-significant differences are displayed as ‘ns’. **(A)** Schematic representation of the experimental set-up to expose seedlings to fungal VOCs. **(B)** Biomass (mean ± SE, *n* = 5–8) of shoots and roots exposed to VOCs produced by fungal mycelium grown on 1/5^th^ Potato Dextrose Agar medium (mycelia_PDA) and on soil-sand mixture (soil_mycelia), and to VOCs produced by fungal sclerotia. **(C)** Time of emergence (mean ± SE, *n* = 8) of radicle and cotyledons of VOC-exposed and control plants. **(D)** Primary root length of VOC-exposed and control plants at 7 days after exposure of *A. thaliana* seeds to fungal VOCs. **(E)** Schematic representation of the experimental set-up to expose seeds and seedlings to fungal VOCs. **(F)** Biomass (mean ± SE, *n* = 5) of shoots and roots of VOC-exposed and control plants at the seed and at the seedling (7 days old) stages. Different letters indicate statistical differences between treatments (One-way ANOVA, Tukey post hoc test, *P* < 0.05).

To investigate the effects of VOCs emitted by the fungal pathogen *R. solani* on the growth and development of *A. thaliana*, 7-day-old seedlings were exposed to the fungal VOCs. To physically separate the plants from the fungus, a three-compartment set-up was used (**Figure 1A**). Sterile *A. thaliana* seeds were sown on Petri dishes (Ø 90 mm) containing 25 mL of half-strength Murashige and Skoog medium (Murashige and Skoog, 1962) supplemented with 5% sucrose (0.5xMS). These Petri dishes (without lids) were kept inside a larger Petri dish (Ø 145 mm) which were sealed and kept in climate cabinets. After 7 days, seedlings were exposed to the fungal VOCs or to agar medium by introducing a small Petri dish (Ø 35 mm) containing a 7-day-old fungal culture (referred to as VOC-exposed) or containing the agar medium or a soil-sand mixture only (control). Petri dishes (Ø 145 mm) were re-sealed and kept in the climate cabinets for 2 weeks. For testing the effect of different substrates on VOC production by the fungus, five different media were used. The media tested included water-agar (HA), water-agar supplemented with sucrose (HS), water-agar supplemented with sucrose and yeast extract (HSY), malt-agar (Oxoid) (Ezra and Strobel, 2003) and 1/5^th^ strength potato dextrose agar (1/5^th^ PDA). Additionally, *R. solani* was grown in a soil-sand mixture. For that, a mycelium plug was added into 2.5 g of sterile soil-sand mixture (12:5 v/v), previously sterilized by autoclaving for 20 min twice with 24 h interval. Fresh and dry weight (overnight incubation at 65°C) of shoots and roots of VOC-exposed plants were determined and compared to control plants. For each treatment, five to six plants per plate were pooled and treated as a single biological replicate, and a total of five to eight biological replicates were used.

To test the effects of fungal VOCs on seed germination and emergence of the radicle and cotyledons, we used two-compartment Petri dishes (Ø 90 mm). One compartment contained 0.5xMS medium and six seeds and the other contained a small Petri dish (Ø 35 mm, without lid) with a 7-day-old fungal culture or 1/5^th^ PDA medium only (control). The two-compartment Petri dish was then closed and incubated as described previously. Seed germination and emergence of cotyledons and radicle were recorded daily. Length of primary root was measured after 7 days of exposure.

**FIGURE 2:**
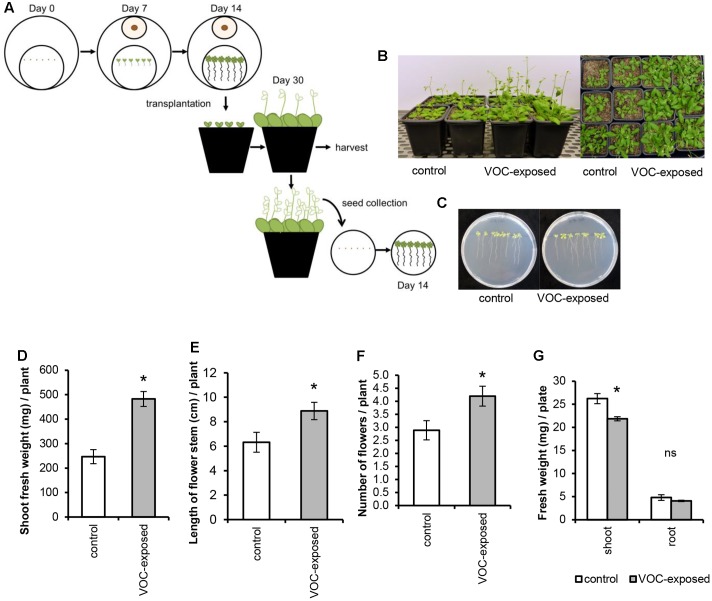
Effects of short exposure of *A. thaliana* to volatile organic compounds (VOCs) produced by *R. solani* on plant growth and development. Plants exposed to fungal VOCs are referred as ‘VOC-exposed’ whereas plants exposed to agar medium are referred as ‘control.’ Asterisks indicate statistically significant differences based on pairwise comparisons between VOC-exposed and control plants (Student’s *t*-test, *P* < 0.05). Non-significant differences are displayed as ‘ns’. **(A)** Schematic representation of the experimental design. **(B)** Representative pictures of *A. thaliana* plants exposed for 1 week to fungal VOCs or not (control) that were transplanted into a soil-sand mixture and grown for 2 weeks in absence of the VOC-producing fungus. **(C)** Pictures of 14-day-old seedlings grown from seeds harvested from VOC-exposed plants. **(D)** Shoot biomass, **(E)** length of flower stem, and **(F)** number of flowers (mean ± SE, *n* = 12) of VOC-exposed and control plants. **(G)** Shoot and root biomass (mean ± SE, *n* = 4) of seedlings grown from seeds harvested from VOC-exposed plants. Each replicate consists of one Petri dish with eight seedlings.

To study the effects of a short exposure to fungal VOCs on plant growth and development, 7-day-old *A. thaliana* seedlings grown *in vitro* were exposed to *R. solani* VOCs (VOC-exposed) or to the agar medium (control) for 1 week as described above and then transferred to pots containing a soil-sand mixture (1:1 v/v) sterilized by autoclaving for 20 min twice with a 24 h interval (**Figure 2A**). Shoot fresh weight, length of floral stem, and number of flowers were determined 2 weeks after soil transplantation. To test if the observed effects of fungal VOCs on plant development could be transferred via the seeds to the next generation (transgenerational effect), 10 VOC-exposed and 10 control plants were kept for seed collection. Seeds were harvested, surface-sterilized, sown in Petri dishes (Ø 90 mm) containing 0.5xMS and grown for 2 weeks under controlled conditions. Fresh and dry weight (overnight incubation at 65°C) of shoots and roots were determined and compared to control plants.

Statistical analyses were performed with IBM SPSS Statistics 23. Data were tested for equal variance using Levene’s test and for normality using Shapiro–Wilk test at the 5% significance level. Statistical differences were determined by pairwise comparisons of VOC-exposed and control plants with Student *t*-test (for independent samples) or One-way ANOVA followed by Tukey’s honestly significant difference (HSD) *post hoc* test. Mann–Whitney *U* test was used when assumptions of normality were not met.

### Effects of CO_2_ on Plant Growth

CO_2_ concentrations are known to be increased in closed systems due to microbial catabolism which, in turn, could enhance photosynthetic efficiency and plant biomass. To investigate potential effects of increased levels of fungal-produced CO_2_ on plant growth, 7-day-old *A. thaliana* seedlings grown on square Petri dishes (Ø 100 mm) were exposed to ambient (400 ppm) and elevated (1330 ppm) CO_2_ levels in 22.4 L desiccators. A total of four to seven replicates were used per treatment. CO_2_ levels were manipulated through mass flow controllers mixing air and CO_2_ to the desired concentrations and monitored with infrared gas analyzers. Desiccators were kept at 20°C; 180 μmol light m^-2^ s^-1^ at plant level; 16 h: 8 h, light: dark. Plant shoot and root biomass was determined after 7 days of exposure. Data were tested for equal variance using Levene’s test and for normality using Shapiro–Wilk test at the 5% significance level. Statistical differences were determined by pairwise comparisons of CO_2_-exposed and control plants with Student *t*-test (for independent samples) using IBM SPSS Statistics 23. Kruskal–Wallis test was used when assumptions of normality were not met.

### Collection and Analysis of Fungal Volatiles

Since also ethylene, a gaseous hormone, is known to be produced by soil microorganisms and to play a role in plant growth and development, we determined the ethylene concentration emitted by *R. solani*. For that, a mycelial plug (Ø 5 mm) was grown in sterile 10 mL glass vials containing 2.5 mL of 1/5^th^ PDA or 0.5xMS media. Vials containing the media only were used as controls. All vials were closed with silicone/PTFE lids and incubated at 25°C for 10 days. Ethylene concentration was measured with a gas chromatograph (Syntech GC 955-100) equipped with a HayeSep 80/100 column and flame-ionization detector as previously described (Pierik et al., 2009). Measurements were performed using five replicates. Statistical differences were determined by pairwise comparisons of treatment and control (medium only) with Mann–Whitney *U* test using IBM SPSS Statistics 23.

To identify other VOCs produced by *R. solani* and potentially involved in the modulation of plant growth and development, fungal cultures were prepared as described for the ethylene measurements. Fungal VOCs were collected by a dynamic headspace system on stainless steel cartridges filled with 200 mg Tenax TA (20/35; Camsco Inc.) as adsorbent. For this, a custom-made ‘needle inlet’ connected to a Tenax cartridge was penetrated through the septum in the lids. VOCs were collected on cartridges by sucking the air out of the vials with a flow of 40 mL min^-1^ for 5 h. A second clean Tenax cartridge was placed similarly to clean the incoming air and to prevent an under pressure. Before thermodesorption, cartridges with the headspace samples were flushed with helium at 50 mL min^-1^ for 5 min to remove moisture and oxygen and after that analyzed by Thermodesorption Gas Chromatography-Mass Spectrometry (TDGC-MS). VOCs were thermally desorbed at 220°C for 7 min (Ultra, Markes Llantrisant, United Kingdom) with a helium flow of 30 mL min^-1^. Analytes were focused at 4°C on a cooled trap (Unity, Markes, Llantrisant, United Kingdom) and were then transferred to the analytical column (ZB-5Msi, 30 m, 0.25 mm i.d., 1.0 μm film thickness, Phenomenex, Torrance, CA, United States) by rapid heating of the cold trap to 260°C for 4 min using a split flow of 5 mL min^-1^. The temperature gradient of the GC oven was as follows: 45°C hold for 3 min, 10°C min^-1^ gradient to 280°C, with a 2 min hold, at a constant gas flow of 1 mL min^-1^. Mass spectra were acquired by electron impact ionization (70 eV) with a scanning from m/z 35-400 with a scan rate of 5 scans s^-1^.

GC-MS raw data were processed by an untargeted metabolomics approach as previously described by Cordovez et al. (2015). VOCs were identified by comparison of the mass spectra with those of authentic reference standards and by spectra of NIST08 (National Institute of Standards and Technology, United States), Wiley libraries and the Wageningen Mass Spectral Database of Natural Products and by comparing the experimentally calculated LRI with the literature values. LRI were calculated based on a series of alkanes using a third order polynomial fitting. Three replicates per treatment were used and vials containing 1/5^th^ PDA medium only were used as controls.

### Plant Exposure to Synthetic Volatiles

To investigate if the fungal compounds detected by GC-MS analysis were responsible for the plant growth-promoting effects, we used single and a mix of synthetic compounds for which authentic reference standards were available. The synthetic compounds 2-methyl-1-propanol (analytical standard), 2-pentanone (99.5%), 2-methyl-1-butanol (≥98.0%), 1-octen-3-ol (98%) and 3-octanone (≥98%) were purchased from Sigma–Aldrich. Experiments were performed in Petri dishes (Ø 90 mm) with two-compartments. Four 7-day-old *A. thaliana* seedlings were placed on one compartment containing 0.5xMS medium and different dilutions of the five synthetic compounds and the mixture were applied to a sterile filter paper (1.5 cm × 1.5 cm) placed in the other compartment. Dilutions of the synthetic compounds were made as previously described by Blom et al. (2011). Briefly, synthetic compounds were diluted with dichloromethane (DCM) to concentrations of 1 ng, 10 ng, 100 ng, 10 μg, 100 μg and 1 mg per 10 μL. Each compound was mixed in a 1:1 ratio with the lanolin solution (1.6 g of lanolin in 10 mL DCM) and 20 μL of this mixture was added to the filter paper. For negative controls, the second compartment was left empty or a mixture of DCM and lanolin was added. For the positive control, seedlings were exposed to the 1-week-old *R. solani* culture grown on a Petri dish (Ø 35 mm) containing 1/5^th^ PDA medium. Petri dishes were immediately sealed and incubated in a growth cabinet. Shoot and root biomass was determined after 2 weeks. Experiments were performed using five biological replicates and statistical differences were determined by pairwise comparison between VOC-exposed and control (exposed to solvent) plants with Student *t*-test (for independent samples) using IBM SPSS Statistics 23. Data were tested for equal variance using Levene’s test and for normality using Shapiro–Wilk test at the 5% significance level.

### Plant RNA Extraction, Sequencing and Transcriptome Analyses

To investigate how VOCs from a soil-borne pathogenic fungus modulate plant growth and development at the transcriptomic level, we sequenced and analyzed the genome-wide transcriptome of *A. thaliana* seedlings exposed to *R. solani* VOCs for 7 days *in vitro*. Seedlings exposed to 1/5^th^ PDA medium only were used as control. For the sequencing of plant RNA, total RNA was extracted from roots and shoots. Four replicates were used, and each replicate consisted of four plates with six seedlings each in order to obtain enough biomass. RNA was obtained from frozen tissues with Trizol reagent (Invitrogen). The RNA samples were further purified using the NucleoSpin RNA II kit (Macherey-Nagel). Samples were processed using the NEBNext Ultra Directional RNA Library Prep Kit for Illumina at ServiceXS (GenomeScan B.V., Leiden, The Netherlands). Briefly, mRNA was isolated from the total RNA suing the oligo-dT magnetic beads. After fragmentation of the mRNA, cDNA was synthesized, ligated with sequencing adapters and amplified by PCR in order to obtain cDNA libraries. Each cDNA library was individually analyzed for quality and yield using a Fragment Analyzer. cDNA was then clustered and a concentration of 1.6 pM was sequenced with an Illumina NextSeq 500 sequencer. Raw data were submitted to the National Center for Biotechnology Information Short Read Archive (SRA BioProject ID: PRJNA392864, SRA sequences: SAMN07313363 to SAMN07313378).

For the transcriptome analysis, Illumina sequences were trimmed and filtered with FASTQC with a threshold of 25 (*Q* > 25). Quality-trimmed reads were counted using RSEM software package (Li and Dewey, 2011) transformed into RPKM (Reads Per Kilobases per Million reads). Reads were mapped to the *A. thaliana* reference genes using the software Bowtie2 v.2.1.0 (Langmead and Salzberg, 2012). The Bioconductor package DESeq2 for R Statistical Analysis (Love et al., 2014) was used for normalization and differential expression analyses. The *P*-value was obtained from the differential gene expression test. FDR (False Discovery Rate) correction was used to determine the *P*-value threshold in multiple tests and analyses. Significant differentially expressed genes (DEGs) were selected using FDR < 0.05 and the absolute value of the log_2_Ratio ≥ 0.585 (at least 1.5× higher than the expression level in control) as thresholds.

Biological interpretation of the DEGs was carried out with a GO-term enrichment analysis performed using AgriGO^1^. TAIR AGI IDs of significantly up- and down-regulated genes were subjected to a singular enrichment analysis using Fisher test with FDR (Hochberg) at 0.05. Arabidopsis TAIR 9 database was used as a background. Identification of transcription factors (TFs) was carried out using the *A. thaliana* TF database^2^.

### Effects of Fungal Volatiles on Plant Resistance to the Fungal Pathogen and Insect Herbivory

To test the effect of a short exposure to fungal VOCs on plant resistance, 1-week-old *A. thaliana* seedlings were exposed to *R. solani* VOCs for 7 days *in vitro* and transplanted to sterile potting soil. After 5 and 14 days, plants were inoculated with the fungal root pathogen *R. solani* or infested with the generalist insect herbivore *M. brassicae*, respectively. To test plant resistance to the fungal pathogen, VOC-exposed plants were inoculated with a mycelial plug (Ø 5 mm) of *R. solani* in the proximity of the roots 5 days after soil transplantation (*n* = 12 pots containing one plant each). Disease incidence was scored after 7, 10, and 14 days post-inoculation (dpi). Leaves were scored as diseased when they exhibited necrotic or chlorotic lesions. Disease incidence was assessed by determining the percentage of diseased leaves per plant (9–12 plants per treatment). Two independent bioassays were performed. A Generalized Linear Model (GLM, Type III Chi-square Wald test) was adopted to statistically assess the effects of VOC-exposure, time point and their interactions on disease incidence. VOC exposure and time point were used as factors. Main effects of VOC exposure and time point as well as the interactions of VOC exposure × time point were analyzed. In addition, the main effects of the interaction of VOC exposure, total number of leaves and time point were tested. Least Significant Difference (LSD) test was used to adjust for multiple comparisons. Statistical analyses were performed using IBM SPSS Statistics 23.

To test plant resistance to insects, VOC-exposed plants were infested with 30 neonates of *M. brassicae* using a fine brush (*n* = 8 pots containing three plants each) 2 weeks after soil transplantation. Plant pots were kept inside a plastic container (Duchefa, Haarlem, The Netherlands; height: 140 mm, upper Ø: 115 mm, lower Ø: 90 mm), covered with insect-proof mesh cloth and sealed with elastic bands and kept in growth cabinet. Caterpillar density was reduced to 15 and 10 larvae at 3 and 7 days post-infestation (dpi), respectively. The reduction of the herbivore number was used to simulate dispersion and predation in nature. Larval performance was measured by weighing the larvae on a microbalance (accuracy = ±1 μg; Mettler-Toledo MT5 Electrobalance) at 3, 7, and 10 dpi. A Generalized Linear Model (GLM, Type III Chi-square Wald test) was performed to statistically assess the effects of VOC-exposure, time, replicate and their interactions on larval weight. VOC exposure, time point and replicate were used as factors. Main effects of VOC exposure, time point and replicate as well as the interactions of VOC exposure × time point and VOC exposure × replicate were analyzed. Bonferroni test was used to adjust for multiple comparisons. Statistical analyses were performed using IBM SPSS Statistics 23.

## Results

### Volatiles from the Fungal Pathogen Promote Plant Growth and Prime Plant Development

To study VOC-mediated interactions, *A. thaliana* seedlings were co-cultivated with, but physically separated from, the fungal pathogen *R. solani* (**Figure 1A**). Seedlings exposed to VOCs from *R. solani* (hereafter referred to as ‘VOC-exposed’) grown on 1/5^th^ PDA medium showed an increase in shoot and root dry weight of 150% (Mann–Whitney *U* test, *P* = 0.008) and 265% (Mann–Whitney *U* test, *P* = 0.008), respectively, compared to seedlings not exposed to the fungal VOCs (hereafter referred to as ‘control’) (**Figure 1B**). Also seedlings exposed to VOCs from fungal mycelium inoculated into a soil-sand mixture showed a 62% increase in root dry weight (**Figure 1B**, *t*-test, *P* = 0.002). We also tested if VOCs emitted from sclerotia, the survival structures of *R. solani* in soil, affect plant growth. Results showed that also sclerotial VOCs increased shoot and root dry weight by 60% (*t*-test, *P* < 0.001) and 44% (*t*-test, *P* = 0.012), respectively (**Figure 1B**). Since the production of VOCs by microorganisms can be influenced by the substrate composition, we tested, in addition to the 1/5^th^ PDA medium, four other growth media with a composition ranging from nutrient-poor to nutrient-rich. For all tested media, except for water agar, dry weights of plant shoots and roots were significantly higher in the fungal VOC-exposed seedlings than in the control (see Supplementary Figure S1).

To study if *R. solani* VOCs can modulate *A. thaliana* development, we investigated their effects on seed germination as well as radicle and cotyledon emergence. Fungal VOCs had no effect on seed germination nor on cotyledon emergence. However, a delay in radicle emergence was observed for VOC-exposed seeds (**Figure 1C**, Mann–Whitney *U* test, *P* = 0.008). Seven days after exposure of the seeds to the fungal VOCs, seedlings displayed shorter primary roots compared to the control (**Figure 1D**; *t*-test, *P* < 0.001). Nevertheless, significant plant growth-promoting effects were observed after 18 days, albeit to a lower extent than observed for seedlings (instead of seeds) exposed to the fungal VOCs (**Figures 1E,F**; ANOVA, *P* < 0.05). To further investigate if *R. solani* VOCs can prime development of plants, *A. thaliana* seedlings were pre-exposed *in vitro* to fungal VOCs for 7 days, transplanted to a soil-sand mixture and grown in absence of the fungus (**Figure 2A**). After 2 weeks, plants pre-exposed to fungal VOCs showed significant increases in shoot weight, in length of the flower stem and in number of flowers of 96, 40, and 45%, respectively (**Figures 2B,D–F**). For 10 VOC-exposed and 10 control plants, seeds were collected and subsequently grown on 0.5xMS agar medium to determine if the VOC-mediated effects on shoot and root growth were transmitted to the next generation of *A. thaliana*. Seedlings originating from seeds of VOCs-exposed plants showed no difference in root weight (*t*-test, *P* = 0.216) and even a slight reduction in shoot weight (*t*-test, *P* = 0.034) compared to seedlings originating from seeds of control plants (**Figures 2C,G**).

Collectively, these results indicate that short- and long-term exposure of different developmental stages of *A. thaliana*, including seed imbibition, to the fungal VOCs significantly enhanced shoot and root biomass, altered root architecture and accelerated development and in particular flowering. The results also suggest that the VOC-mediated effects do not seem be transgenerational although more generations need to be tested to further support this finding.

### Identification of Fungal Volatiles and Their Effects on Plant Growth

Previous studies have reported increased CO_2_ concentrations in closed systems due to microbial catabolism. Higher CO_2_ concentrations may lead to enhanced photosynthetic efficiency and increased plant biomass (Poorter and Navas, 2003; Kai and Piechulla, 2009; Jauregui et al., 2015). To evaluate a role of CO_2_ in the observed plant growth-promoting effects by *R. solani* VOCs, our first approach was to trap CO_2_ with different amounts of the CO_2_ absorber, sodalime. The results showed that even small amounts of sodalime were harmful to the growth of *A. thaliana* seedlings, suggesting a deleterious depletion of CO_2_ (see Supplementary Figure S2A). A second approach involved exposing the seedlings to elevated CO_2_ concentration of 1330 ppm, which is approximately three times higher than the ambient CO_2_ concentration of 400 ppm. After 7 days of plant growth, no significant differences in plant shoot (*t*-test, *P* = 0.219) and root (Kruskal–Wallis test, *P* = 0.389) weights were observed between CO_2_-exposed and control seedlings (see Supplementary Figures S2B,C).

**FIGURE 3:**
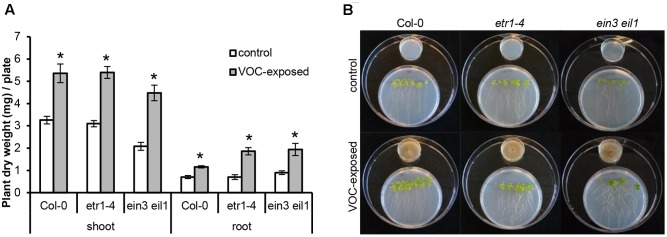
Effects of volatile organic compounds (VOCs) from *R. solani* on the growth of ethylene-insensitive mutants of *A. thaliana*. **(A)** Shoot and root biomass (mean ± SE, *n* = 4–5) of *A. thaliana* wild-type Col-0 and mutants *etr1-4* and *ein3 eil1* exposed to VOCs from *R. solani*. Each replicate consists of one Petri dish with six seedlings. Asterisks indicate a statistically significant difference between VOC-exposed and control (exposed to agar medium only) plants (Student’s *t*-test, *P* < 0.05). **(B)** Phenotype of *A. thaliana* wild-type and mutants after 14 days of exposure to the fungal VOCs.

To determine if ethylene, a volatile hormone produced by several microorganisms (Ilag and Curtis, 1968; Considine et al., 1977), was involved in the plant growth-promoting effects, we first determined if *R. solani* produced ethylene under the growth conditions used in the bioassay. Among the VOCs emitted by a 10-day-old fungal culture, no differences in ethylene concentration were found relative to the control (see Supplementary Figure S3). Since we cannot exclude that ethylene production occurs at earlier or later growth stages of *R. solani* or during its interaction with plants, we tested two independent ethylene-insensitive *A. thaliana* mutants for their response to the fungal VOCs. These mutants were *etr1–4* (ethylene receptor mutant) and *ein3 eil1* (TF double mutant). The results showed that VOC-mediated plant growth promotion was observed for both ethylene-insensitive mutants and to the same extent as observed in wild-type *A. thaliana* Col-0 (**Figures 3A,B**). In these assays, we also included *Verticillium dahliae*, a soil-borne pathogenic fungus that produces ethylene when grown on 1/5^th^ PDA. *V. dahliae* still promoted plant growth in the two ethylene-insensitive *A. thaliana* mutants (data not shown). Collectively, these results indicate that ethylene does not play a major role in the plant growth promotion by *R. solani* VOCs observed in our experimental system.

Subsequent GC-MS analysis revealed a total of 14 VOCs in the headspace of *R. solani* cultures that were significantly different from the control (agar medium only) (*t*-test, *P* < 0.05) and with peak intensities at least twice as high as those in the control (**Table 1**). Among these, we detected the typical fungal VOCs such as 1-octen-3-ol and 3-octanone. Five VOCs, for which authentic reference standards were available, were tested for their effects on plant growth. When *A. thaliana* seedlings were exposed to 2-methyl-1-propanol, 2-pentanone, 2-methyl-1-butanol, 1-octen-3-ol or 3-octanone at concentrations ranging from 1 ng to 1 mg per 10 μl or a mix of all five synthetic volatile compounds, no growth promotion was observed compared to seedlings exposed to the solvent only, whereas VOCs from *R. solani* showed a significant growth promotion (see Supplementary Figure S4A). Higher concentrations (10 μg, 100 μg and 1 mg) of 1-octen-3-ol, 3-octanone and the mix even inhibited plant growth (see Supplementary Figure S4B). These results indicate that other concentrations or other mixtures of the tested VOCs, or other VOCs not detected by the analytical method used here are involved in plant growth promotion by *R. solani*.

**Table 1:** Volatile organic compounds (VOCs) emitted by the fungal pathogen *Rhizoctonia solani*. VOCs displayed are significantly different (Student’s *t*-test, *P* < 0.05, *n* = 3), being at least twice as abundant as in the control (medium only). Compounds were identified by comparing their mass spectra and LRI with those of authentic reference standards (MSI level 1) or with spectra and LRI published in the NIST08 and in-house mass spectral libraries (MSI level 2).

			VOC emission	
			
Compound	RI^a^	Annotation^b^	control	*R. solani*
2-methyl-1-propanol^∗^	665	1	258225 ± 59955	739090 ± 106560
Unknown	706	4	3664 ± 1222	318349 ± 121863
2-pentanone^∗^	709	1	145709 ± 18534	329667 ± 47446
Methyl thiocyanate	732	2	17812 ± 8687	583890 ± 36242
2-methylbutanenitrile	740	1	24682 ± 18783	414931 ± 118132
3-methyl-butanenitrile	745	1	72109 ± 11955	1169000 ± 273882
2-methyl-1-butanol^∗^	751	1	33574 ± 4041	470300 ± 181198
Unknown	956	4	11654 ± 10118	30769 ± 5295
1-octen-3-ol^∗^	978	1	4620 ± 2684	2255670 ± 1373443
3-octanone^∗^	986	1	12785 ± 10514	3549774 ± 1893459
2-ethyl-1-hexanol	1027	2	264835 ± 59710	507060 ± 88147
2-nonen-1-ol	1172	1	1956 ± 281	4839 ± 1900
Unknown	1343	4	3468 ± 869	5971 ± 1257
Alpha-copaene	1403	2	5092 ± 3553	45013 ± 21268


*^a^Calculated retention indices (RI).*

*^b^Annotation level according to the Metabolomics Standards Initiative (MSI).*

*^c^Emission values refer to peak intensity (mean ± SD).*

*^∗^Compounds tested for plant-growth promoting effects.*

### Fungal Volatiles Induce Plant Genome-Wide Transcriptional Changes

**FIGURE 4:**
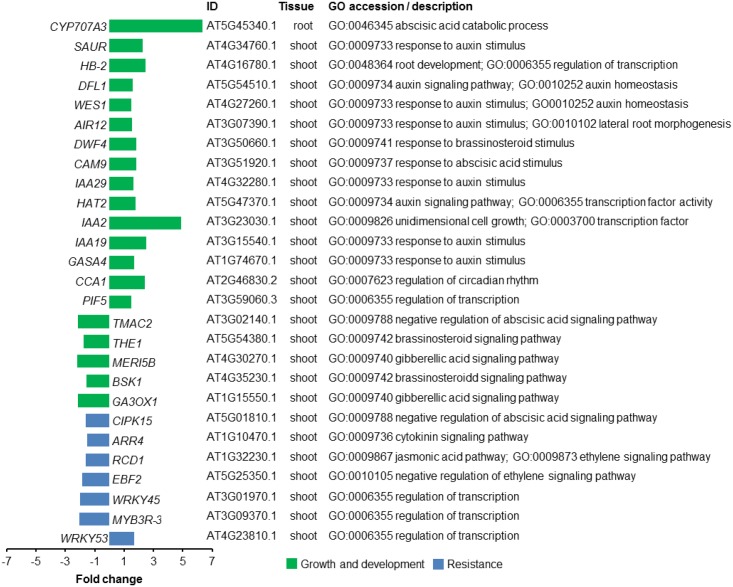
Transcriptional changes in genes of *A. thaliana* exposed to volatile organic compounds (VOCs) from *R. solani.* Differentially expressed genes (DEGs) for seedlings exposed to the fungal VOCs are shown. Genes described to be involved in plant growth and development are depicted in green, whereas genes associated with resistance responses to biotic stress are depicted in blue. Gene Ontology (GO) description is based on the output from AgriGO.

To begin to understand the molecular mechanisms underlying plant growth promotion by VOCs from the fungal root pathogen *R. solani*, we performed genome-wide transcriptome analysis. Significant DEGs were selected with a log_2_ fold change ≥ +0.058 (1.5× higher) or ≤-0.585 (1.5× lower) and FDR < 0.05. Following these criteria, a total of 477 (267 up-regulated and 210 down-regulated) and 12 (6 up-regulated and 6 down-regulated) genes were identified in *A. thaliana* shoot and root tissues exposed to *R. solani* VOCs, respectively (see Supplementary Table S1). To identify enriched Gene Ontology (GO) terms, a singular enrichment analysis (SEA) of GO categories was conducted using AgriGO (see Supplementary Tables S2, S3). Transcriptional changes in genes involved in growth and resistance of *A. thaliana* are highlighted in **Figure 4**.

Among the GO terms identified for shoot up-regulated genes, we found an enrichment of genes involved in photosynthesis and auxin response. Since plant hormones have an essential role in integrating plant growth, development and resistance, we zoomed in on the GO-term ‘hormone-mediated signaling pathway’ to further investigate how fungal VOCs affect the balance of these processes in the *A. thaliana* plants. Up-regulated shoot genes in this category included genes involved in auxin and abscisic acid mediated (ABA) signaling pathways. Genes involved in auxin homeostasis included *IAA-2* (AT3G23030.1), *IAA-19* (AT3G15540.1), *IAA-29* (AT4G32280.1), *WES1* (AT4G27260.1), *HAT2* (AT5G47370.1), and *DFL1* (AT5G54510.1) (**Figure 4**). Also, other genes involved in the response to auxin, such as *PIF5* (AT3G59060) and *HB-2* (AT4G16780.1), were found among the up-regulated genes. In addition, *CYP707A3* (AT5G45340.1), involved in ABA catabolic process, was 6.3-fold up-regulated in root genes of VOC-exposed seedlings. Down-regulated shoot genes included genes involved in ethylene and jasmonic acid (JA) signaling pathways. Genes involved in response to gibberellin (GA) and brassinosteroid (BR) stimuli were both up- and down-regulated in shoot tissue (see Supplementary Tables S4, S5). It was not possible to perform an enrichment analysis for root DEGs since the total number of DEGs was lower than the required minimum number of genes in the query. Given the up-regulation of genes involved in the response to auxin, we tested a number of *A. thaliana* mutants impaired in auxin perception (*tir1 afb1*), biosynthesis (*wei8*), the PHYTOCHROME INTERACTING FACTOR (*pif4 pif5*) as well as an overexpressor of a stable version of the negative regulator of PIF activity HFR1 (*G-BH-03*) and the *hfr1* knockout mutant. The results showed that VOC-mediated plant growth promotion was observed for all mutants to a similar or greater extent as observed for wild-type *A. thaliana* Col-0 (see Supplementary Figure S5). These results suggest that the PIF-auxin axis of growth regulation does not play a major role in the growth-promoting effects of *A. thaliana* seedlings exposed to *R. solani* VOCs.

To study changes in the expression of regulatory genes of *A. thaliana* induced by *R. solani* VOCs, we also identified TFs encoded by the DEGs using a web-based tool^3^. We found a total of 20 and 22 TFs that were up- and down-regulated, respectively, when seedlings were exposed to the fungal VOCs (see Supplementary Table S6). Previous studies with *A. thaliana* have demonstrated that members of the WRKY and the MYB TF families control various processes involved in the responses to biotic and abiotic stresses, development, metabolism, and defense. Our analyses identified several members of the MYB and the WRKY families: *WRKY53* (AT4G23810) was up-regulated, whereas *WRKY26* (AT5G07100), *WRKY39* (AT3G04670), *WRKY45* (AT3G01970), and MYB3R-3 (AT3G09370) were down-regulated in plants exposed to fungal VOCs. Some of these down-regulated TFs have been described for their role in plant resistance to abiotic stresses, such as *WRKY26* and *WRKY39*, and to biotic stresses, such as *WRKY45* (Li et al., 2011; Shimono et al., 2012; Cheng et al., 2015).

### Fungal Volatiles Affect Plant Resistance to Insect Herbivores But Not to the VOC-Producing Fungal Pathogen

**FIGURE 5:**
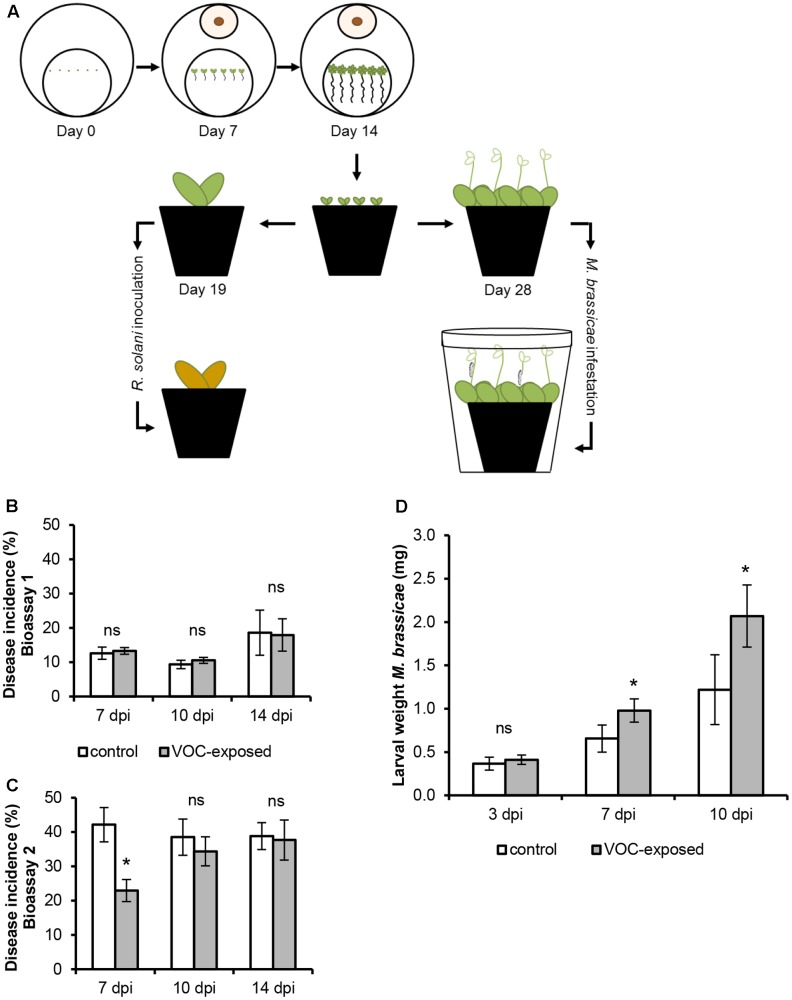
Effects of volatile organic compounds (VOCs) from *R. solani* on resistance of *A. thaliana* to fungal infection and insect herbivory. **(A)** Schematic representation of the experimental set-up to test plant resistance to the fungal pathogen and the insect herbivore. **(B,C)** Disease incidence (mean ± SE, *n* = 9–12) of VOC-exposed *A. thaliana* challenged with *R. solani* obtained in two independent bioassays. Disease symptoms were determined at 7, 10, and 14 days post inoculation (dpi). **(D)** Biomass of *Mamestra brassicae* larvae (mean ± SE, *n* = 8) feeding on *A. thaliana* previously exposed to fungal VOCs or to the agar medium only (control). Each pot contained three plants which were infested with 30 neonates. Larval weight was measured at 3, 7, and 10 days post-infestation (dpi). Asterisks indicate statistically significant differences based on pairwise comparisons between VOC-exposed and control plants (GLM, *P* < 0.05). Non-significant differences are displayed as ‘ns.’

To determine if VOCs can modulate disease resistance, we investigated if plants that were previously exposed to the VOCs emitted by *R. solani* had become more resistant to infection by *R. solani* itself (**Figure 5A**). The overall results of two independent bioassays showed that *R. solani* disease incidence of VOC-exposed plants, monitored at different time points, did not differ from that of the control plants (**Figure 5B**; GLM, *P* = 0.836). In the second bioassay, disease incidence at 7 dpi was significantly lower in VOC-exposed plants than in control plants leading to a significant overall effect of the VOC exposure on disease incidence (**Figure 5C**; GLM, *P* = 0.025). It should be emphasized, however, that the absolute number of leaves with disease symptoms was similar for control and VOC-exposed plants, however, the increased number of leaves in VOC-exposed plants (see Supplementary Figure S6) resulted in a lower disease incidence. No significant interaction effects were found between the VOC exposure, the total number of leaves and the time points (GLM, *P*_bioassay_1_ = 0.101, *P*_bioassay_2_ = 0.139).

To further investigate the effect of fungal VOCs on aboveground resistance, we exposed plants to the insect herbivore *M. brassicae* (**Figure 5A**). Biomass of larvae feeding on leaves of fungal VOC-exposed plants increased in fresh weight by 49 and 70% at 7 and 10 dpi, respectively, as compared to that of larvae feeding on leaves of non-exposed control plants (**Figure 5D**). Statistically significant effects of the time point (GLM, *P* ≤ 0.001), VOC exposure (GLM, *P* ≤ 0.001), replicate (GLM, *P* = 0.005) as well as the interactions between VOC exposure and time point (GLM, *P* ≤ 0.001) and between VOC exposure and replicate (GLM, *P* = 0.001) were observed. These analyses indicate that exposure of *A. thaliana* to *R. solani* VOCs did not affect belowground resistance to the VOC-producing fungus itself but reduced aboveground resistance to insect larval feeding.

## Discussion

Soil microorganisms can affect plant growth and disease resistance from a distance via the production of VOCs, however, the natural functions, the chemistry and underlying molecular mechanisms are largely unknown. Here, we showed that VOCs from the fungal root pathogen *R. solani* led to an increase in plant biomass, an effect that confirms and extends recent results obtained for plant pathogenic *Fusarium* and *Alternaria* species (Bitas et al., 2015; Sanchez-Lopez et al., 2016). No plant growth promotion was observed for five *R. solani* VOCs tested individually and as mixtures, suggesting that other mixtures of the tested VOCs or other VOCs not detected are involved in plant growth promotion by *R. solani*.

The results of this study showed, for the first time, that *R. solani* VOCs primed and accelerated the development of *A. thaliana* seedlings leading to a higher number of flowers in VOC-exposed plants. These findings suggest that plants sensing the presence of the fungal root pathogen via VOCs accelerate their development to produce offspring faster. Hence, we hypothesized that VOCs emitted by the pathogenic fungus may function as an ‘alert’ signal to plants, accelerating growth and development as well as triggering plant resistance in case of a physical encounter with the pathogen. Alternatively, plants may use enhanced growth upon perception of a potential pathogen as a defensive strategy, sacrificing part of the root or shoot biomass and reallocating resources into reproduction rather than in defense processes. Our results showed that exposure of *A. thaliana* to the VOCs from *R. solani* did alter resistance to herbivory aboveground but did not affect resistance belowground to infections by the VOC-producing fungal pathogen itself. Based on these results, we postulate that the fungal pathogen may use VOCs to manipulate, from a distance, plant growth and metabolism to its own benefit. The VOC-induced increase in root biomass and lateral root formation provides a greater root surface area for fungal colonization and infection. Especially for immobile sclerotia, these phenotypic changes in root biomass and architecture would increase the chances of a physical encounter with the roots of host plants.

Previous studies have shown that fungal metabolites can manipulate its host plants to favor invasion and nutrient uptake as well as to counteract the establishment of host immunity (Biemelt and Sonnewald, 2006; Chanclud and Morel, 2016). For example, *Tuber* species have been shown to manipulate ethylene pathways in its host by the production of this hormone, inducing root morphological modifications (Splivallo et al., 2009). In our study, no evidence was found for a role of ethylene in the phenotypic responses induced in *A. thaliana* by *R. solani* VOCs. In addition to hormones, oxylipins, have been proposed as developmental and communication signals between fungi and plants (Tsitsigiannis and Keller, 2007). In plants, oxylipins are involved in the regulation of plant growth and development as well as defense to biotic and abiotic stresses, whereas in fungi they are used as a developmental signal for fungal spore germination and growth (Brodhun and Feussner, 2011). Several lines of evidence suggest that plant oxylipins can be partly substituted for fungal oxylipins and that fungal oxylipins can influence plant development by mimicking the plant endogenous signal communication (Tsitsigiannis and Keller, 2007; Brodhagen et al., 2008). VOC profiling of *R. solani* showed the presence of the oxylipins 1-octen-3-ol and 3-octanone, but these and other VOCs did not show a significant effect on plant growth when tested individually or in a mixture; several were even toxic at higher concentrations.

To begin to understand the molecular mechanisms underlying VOC-mediated interactions between *A. thaliana* and *R. solani*, we looked into the transcriptional changes triggered by plant exposure to the fungal VOCs. Our genome-wide transcriptome analysis revealed that *R. solani* VOCs modulated the expression of genes involved in auxin and ABA responses as well as photosynthesis. Among the up-regulated genes, we observed an enrichment of the genes involved in auxin homeostasis such as *IAA2*, *IAA19*, *IAA29*, *HAT2*, *DFL1*, and *WES1*. Auxin is a common target for the manipulation of plant development and resistance by either beneficial or pathogenic microorganisms (Grunewald et al., 2009; Cui et al., 2013). This hormone stimulates plant growth which can then be exploited by these microorganisms. In addition, auxin can also favor pathogen infection, for example, by increasing expansin production which in turn facilitates penetration of the plant cell wall (Pusztahelyi et al., 2015). In a recent study, Garnica-Vergara et al. (2016) showed that auxin signaling in *A. thaliana* is affected by the volatile 6-pentyl-2H-pyran-2-one emitted by the beneficial fungus *T. atroviride*. Also Bitas et al. (2015) showed that *F. oxysporum* can manipulate auxin transport and signaling via the production of VOCs. Although the results from our transcriptome analyses also pointed to modulation of auxin in seedlings exposed to the VOCs from *R. solani*, several *A. thaliana* mutants in auxin perception, biosynthesis and signaling still showed an increase in plant growth when exposed to the fungal VOCs. Thus, it is likely that the mechanisms underlying the plant growth-promoting effect by *R. solani* are mediated via other signaling pathways not detected in our transcriptome analysis. Sanchez-Lopez et al. (2016) have shown that VOCs emitted by *A. alternata* changed the expression of light- and CK-responsive genes involved in photosynthesis, growth and flowering. The authors concluded that the fungal VOCs promoted plant growth and flowering through cytokinin signaling. Although *R. solani* VOCs also altered genes involved in photosynthesis, we were not able to detect an enrichment of genes involved in cytokinin signaling. Extensive temporal transcriptome analyses of plants exposed to fungal VOCs will be needed to get more detailed insight into the critical time points in the trajectory of plant development, where the fungal VOCs prime essential transcriptional changes that lead to plant growth promotion.

Interestingly, several of the genes found to be up-regulated in VOC-exposed plants, in particular *IAA2*, *IAA19*, *IAA29*, and *HAT2*, have been reported to be associated with shade-avoidance responses (de Wit et al., 2014). When competing for light, *A. thaliana* plants invest in shade-avoidance responses such as increased leaf angles, stem elongation, apical dominance and early flowering, phenotypes that allow competition with neighboring plants (Casal, 2013; Ballaré and Pierik, 2017). Our results further show the up-regulation in shoot tissues of *PIF5*, a phytochrome interacting-factor also involved in shade-avoidance responses. *PIF5* regulates elongation growth by controlling the expression of genes that code for auxin biosynthesis and auxin signaling components (Lorrain et al., 2008; Hornitschek et al., 2012). Plant investment in growth during shade-avoidance responses compromises pathogen resistance by repression of JA-dependent defense mechanisms (de Wit et al., 2013). Although VOC exposure showed no change in belowground plant resistance against the fungal pathogen *R. solani*, it did show a negative effect on aboveground resistance to the insect herbivore *M. brassicae*.

## Conclusion

Plant growth promotion by microbial VOCs appears to be a widespread phenomenon, not only observed for beneficial microorganisms but also for fungal root pathogens. Our results indicate that VOCs from a soil-borne pathogenic fungus can prime plant growth and development thereby influencing the trade-off between plant growth/development and resistance to insect herbivores. VOC-mediated interactions are dependent on the dynamics of the whole ecosystem including soil properties and the complex and competitive interactions among (micro)organisms. How the VOC-mediated phenotypic changes in plant growth affect plant chemistry and tri-trophic interactions is yet unknown. Future investigations will further expand on this line of research and focus on effects of microbial VOCs, both from saprophytic and pathogenic soil-borne fungi in their natural habitat. These studies will further contribute to a better understanding of the natural roles of microbial VOCs in the plant root-soil interface.

## Author Contributions

VC designed and performed the experiments, carried out the data analyses and drafted the manuscript. LM assisted with the experimental design, CO_2_ measurements and CO_2_ exposure assays. KM and DL-B assisted with the insect resistance assay and data analysis. RP carried out the fungal ethylene measurements, assisted with CO_2_ exposure assays and provided *A. thaliana* mutants. RM assisted with the fungal VOC collection and analysis. VJC assisted with experimental design and transcriptome analyses. JR coordinated the study and assisted with the experimental design and with drafting the manuscript. All authors agreed with the final version of the manuscript.

## Conflict of Interest Statement

The authors declare that the research was conducted in the absence of any commercial or financial relationships that could be construed as a potential conflict of interest.

## Footnotes

1 http://bioinfo.cau.edu.cn/agriGO/index.php

2 http://arabidopsis.med.ohio-state.edu/

3 http://arabidopsis.med.ohio-state.edu/AtTFDB
