# Supplementary material for: Plant Phenotypic and Transcriptional Changes Induced by Volatiles from the Fungal Root Pathogen Rhizoctonia solani
Source: Front Plant Sci. 2017 Jul 21;8:1262. doi: 10.3389/fpls.2017.01262 (PMC5519581; doi:10.3389/fpls.2017.01262)
Supplement: Supplementary file 7 [file Presentation_1.PDF]

Supplementary Material

Plant phenotypic and transcriptional changes induced by volatiles from the fungal root pathogen *Rhizoctonia solani*

Viviane Cordovez[1,2], Liesje Mommer[3], Kay Moisan[1,4], Dani Lucas-Barbosa[4], Ronald Pierik[5], Roland Mumm[6,7], Victor J. Carrion[1], Jos M. Raaijmakers[1,8*]

*** Correspondence:** Prof. dr. Jos M. Raaijmakers: j.raaijmakers@nioo.knaw.nl

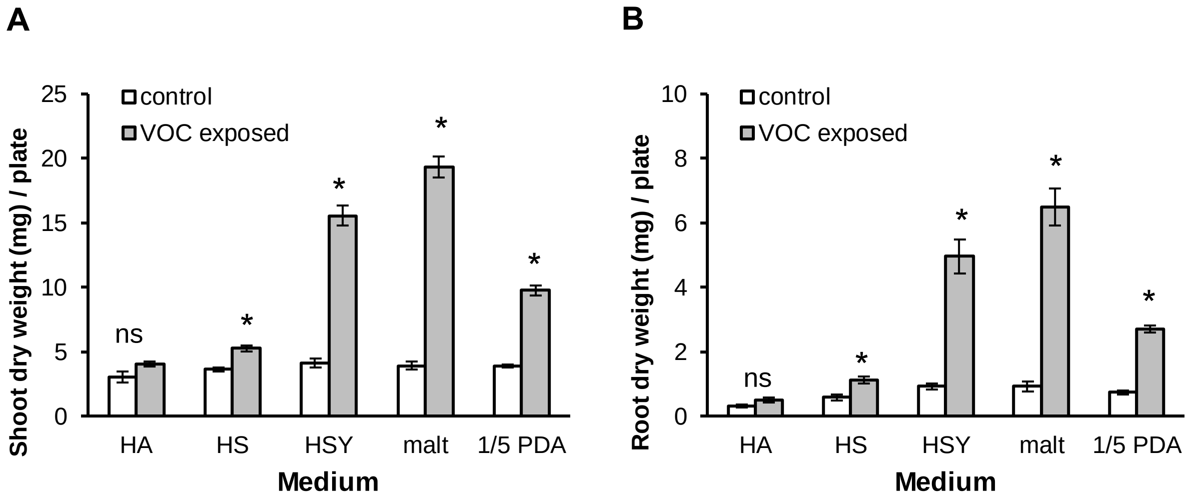

Supplementary Figure 1. Biomass of *Arabidopsis thaliana* shoots **(A)** and roots **(B)** exposed to volatile organic compounds (VOCs) emitted by *Rhizoctonia solani* grown on different media (HA: water agar, HS: agar-water with sucrose, HSY: water-agar with sucrose, HSY: yeast extract, malt-agar and 1/5th PDA: 1/5th potato dextrose agar). Data represent the mean ± SE (n = 4). Asterisks indicate statistically significant differences between VOC-exposed and control (exposed to agar media only) plants (Student's t-test, $P < 0.05$). Non-significant differences are displayed as 'ns'.

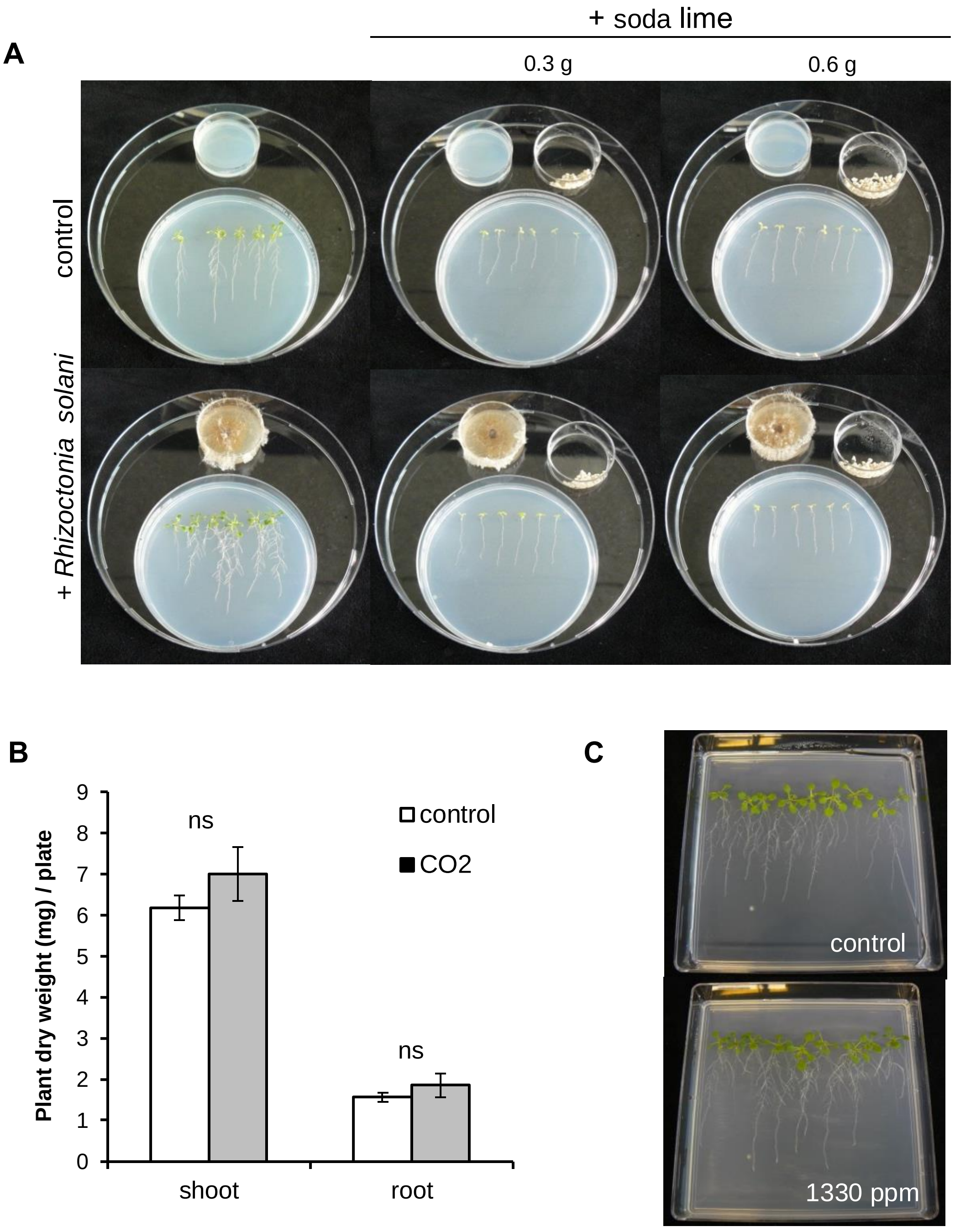

Supplementary Figure 2. (A) Effects of different concentrations of sodalime on *Arabidopsis thaliana* growth in the presence and absence of *Rhizoctonia solani*. **(B)** *A. thaliana* shoot and root biomass (mean ± SE, n = 4) after exposure of 1330 ppm CO_2. 'n.s.' indicates no statistically significant difference between CO_2-exposed and non-exposed seedlings (Student's t-test, $P < 0.05$). **(C)** Pictures were taken after 14 days CO_2 exposure.

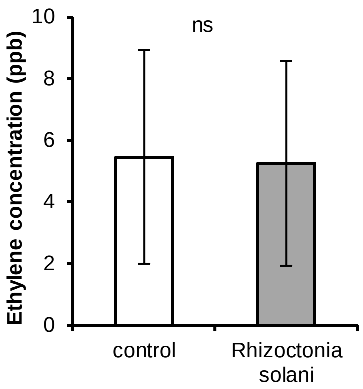

Supplementary Figure 3. Production of ethylene by *Rhizoctonia solani*. Gas-chromatographic measurement of ethylene (mean ± SE, n = 4-5) emitted by 7-day-old culture of *Rhizoctonia solani* grown on $1/5^{th}$ Potato Dextrose Agar medium. Control refers to medium only and non-significant difference is displayed as 'ns' (Mann-Whitney *U* test, $P < 0.05$).

A

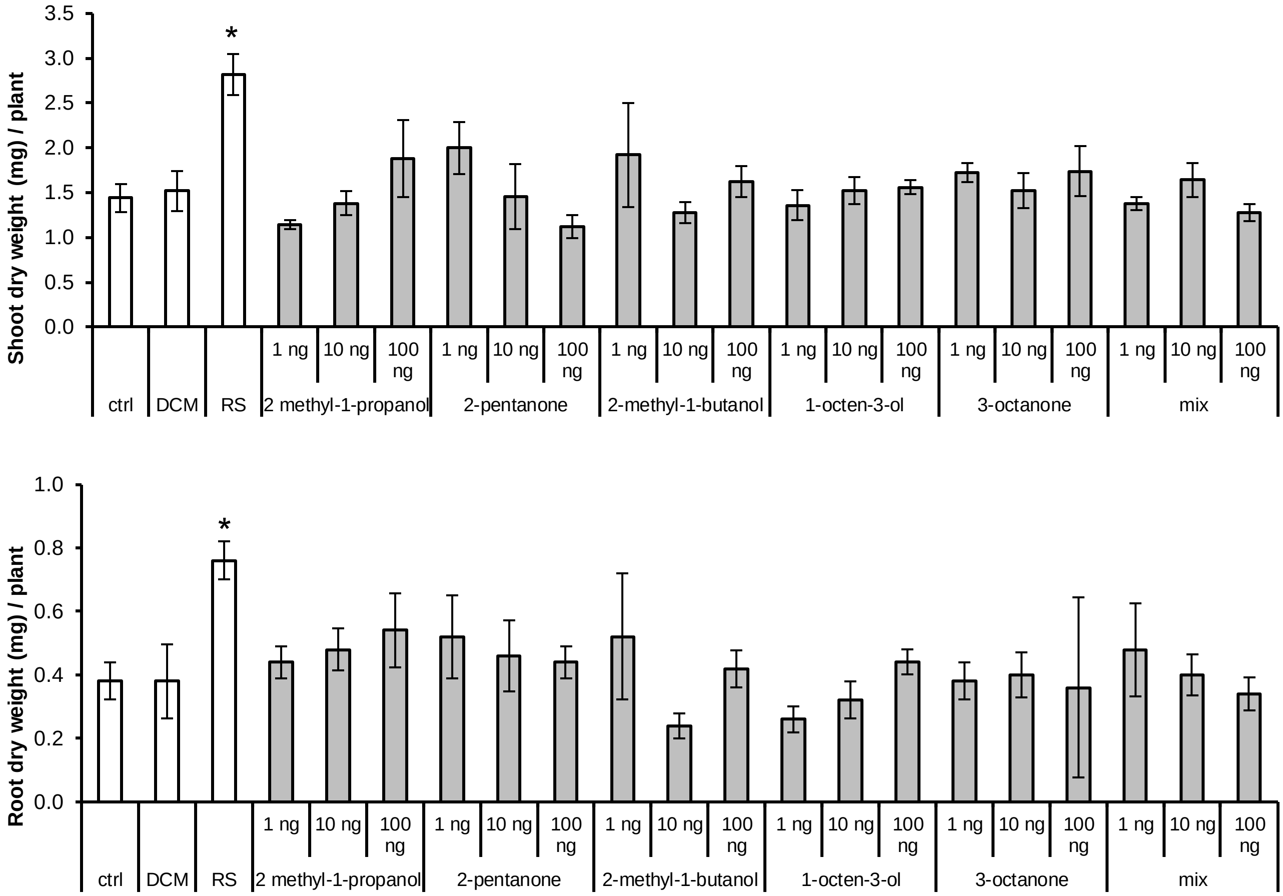
Shoot dry weight (mg) / plant
3.5
3.0
2.5
2.0
1.5
1.0
0.5
0.0
*
1 ng
10 ng
100 ng
ctrl
DCM
RS
2 methyl-1-propanol
2-pentanone
2-methyl-1-butanol
1-octen-3-ol
3-octanone
mix
Root dry weight (mg) / plant
1.0
0.8
0.6
0.4
0.2
0.0
*

B

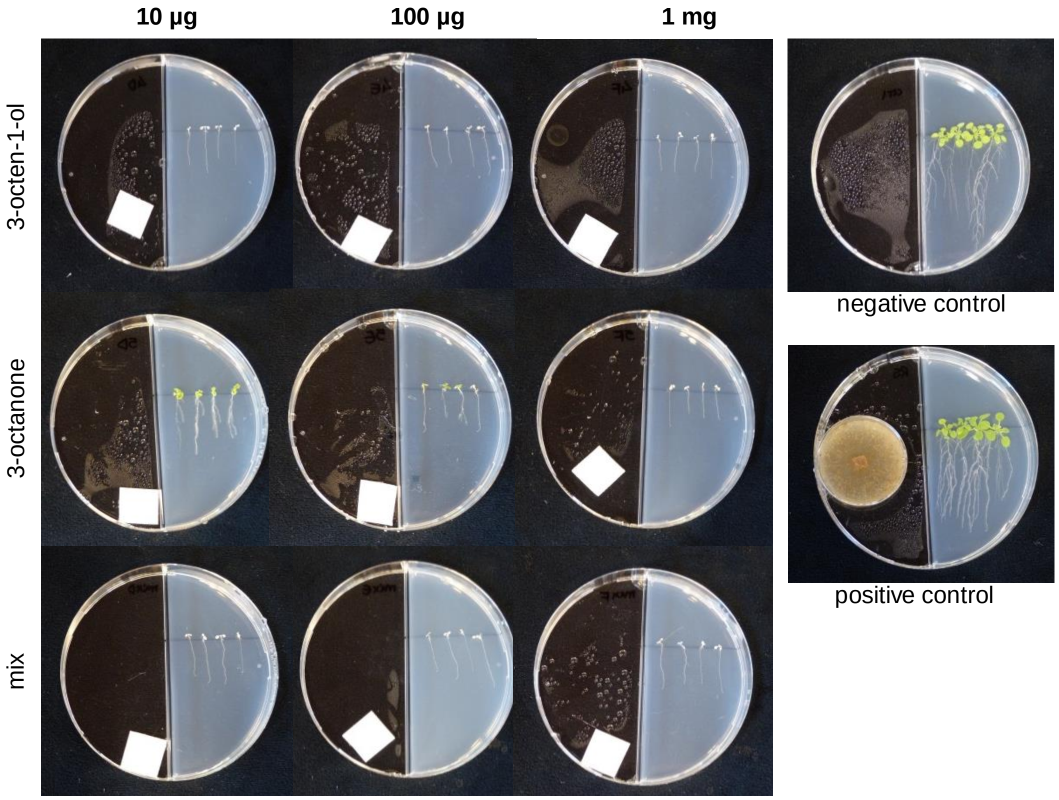
10 µg
100 µg
1 mg
3-octen-1-ol
3-octanone
mix
negative control
positive control

Supplementary Figure 4. Effects of synthetic volatile compounds (VOCs) on plant growth. **(A)** Shoot (top) and root (bottom) biomass (mean ± SE (n = 5) of *Arabidopsis thaliana* exposed to concentrations of 1, 10 and 100 ng of 2-methyl-1-propanol, 2-pentanone, 2-methyl-1-butanol, 1-octen-3-ol, 3-octanone and the mixture of all five VOCs. A mixture of dichloromethane and lanolin (DCM) was used as solvent. Plants exposed to *Rhizoctonia solani* mycelia were used a positive control (RS). Asterisks indicate statistically significant differences as compared to control (exposure to DCM) (Student's t-test, $P < 0.05$). **(B)** Phytotoxic effects of 3-octen-1-ol, 3-octanone and the mix at high concentrations (10 µg, 100 µg and 1 mg) after 14 days of exposure.

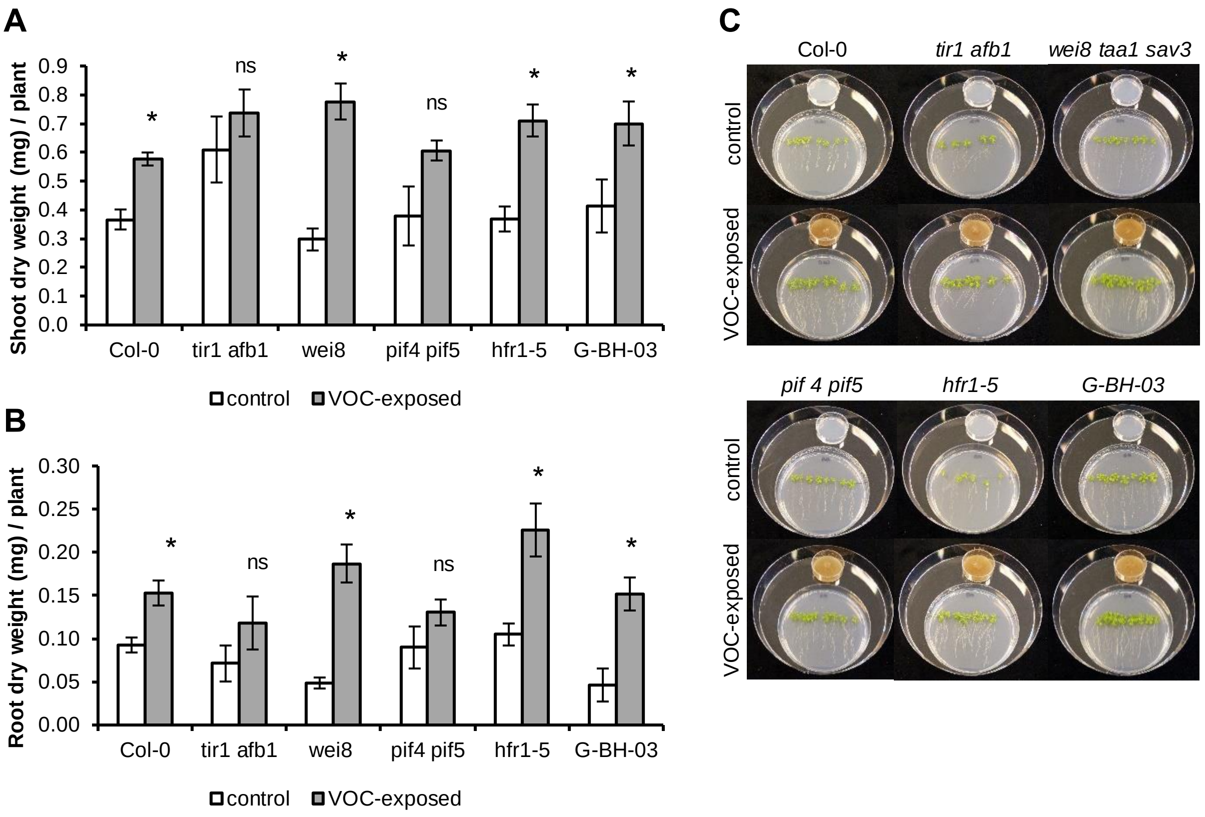

Supplementary Figure 5. Effects of volatile organic compounds (VOCs) emitted by *Rhizoctonia solani* on the growth of *Arabidopsis thaliana* auxin mutants. Shoot **(A)** and root **(B)** biomass (mean ± SE, n = 3-6) of *A. thaliana* wild-type Col-0 and the mutants *tir1afb1*, *wei8*, *pif4pif5*, *hfr1-5* and *G-BH-03* VOCs emitted by *R. solani*. Asterisks indicate a statistically significant difference between VOC-exposed and control (exposed to agar medium only) plants (ANOVA, $P < 0.05$). Non-significant differences are displayed as 'ns'. **(C)** Phenotype of *A. thaliana* wild-type and mutants after 14 days of exposure to fungal VOCs.

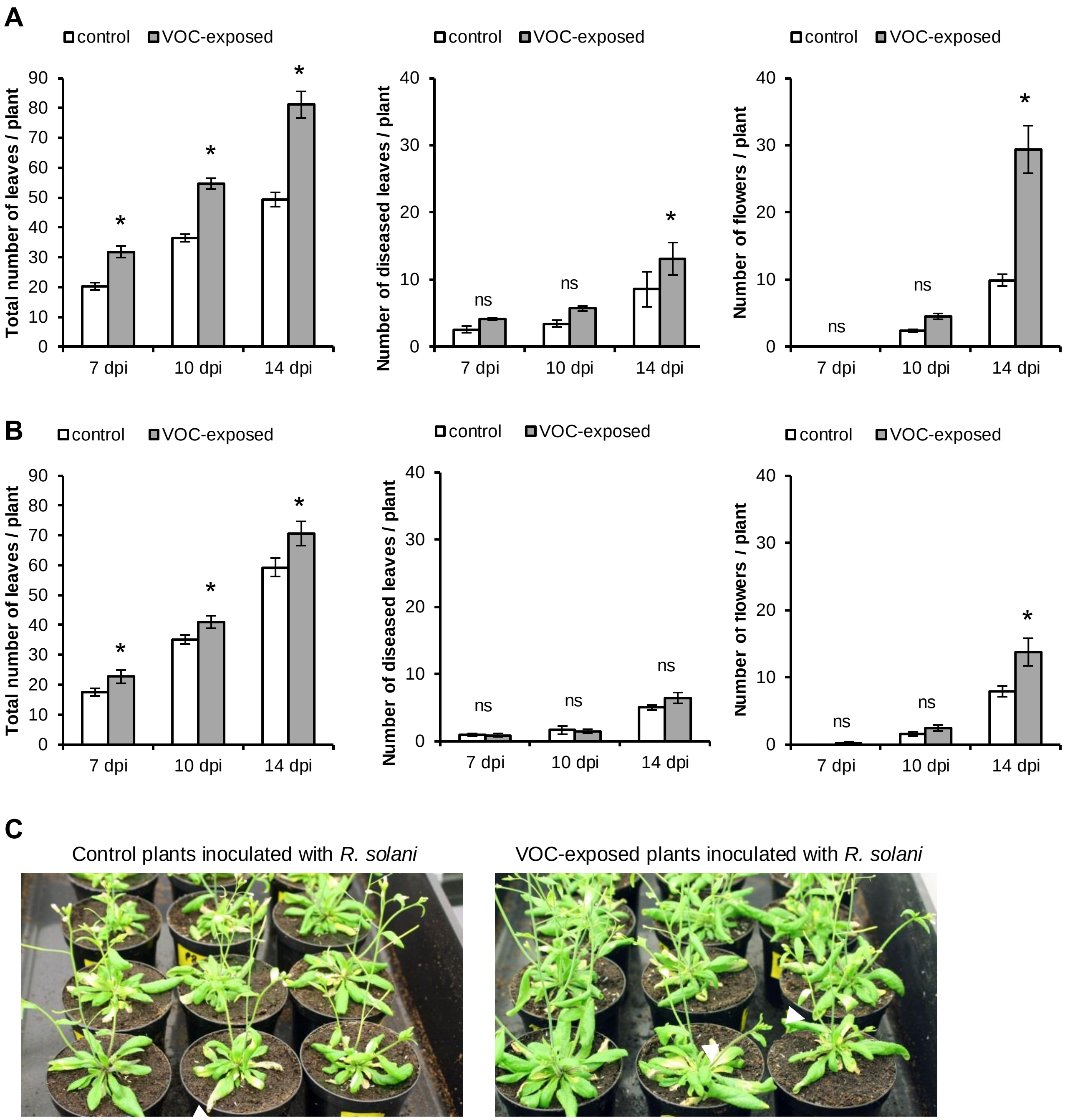

Supplementary Figure 6. Effects of volatile organic compounds (VOCs) emitted by *Rhizoctonia solani* on *Arabidopsis thaliana* resistance to infection by *R. solani*. (A) Bioassay 1: total number of leaves, absolute number of diseased leaves and number of flowers per plant (mean ± SE, n = 9) observed at 7, 10 and 14 days post inoculation (dpi) of the fungal pathogen. **(B)** Bioassay 2: total number of leaves, absolute number of diseased leaves and number of flowers per plant (mean ± SE, n = 12) observed at 7, 10 and 14 days post inoculation (dpi) of the fungal pathogen. Asterisks indicate statistically significant differences resulted from pairwise comparisons between VOC-exposed and control plants (GLM, $P < 0.05$). Non-significant differences are displayed as 'ns'. **(C)** Disease symptoms observed for control (left) and VOC-exposed plants inoculated with *R. solani*. Arrows indicate chlorotic leaves. Pictures were made at 14 dpi.
